# Moving from change agents to improvement architects through the Four Cs model: rethinking healthcare's complexity, cultures, contexts and connections

**DOI:** 10.3389/frhs.2026.1687699

**Published:** 2026-06-02

**Authors:** Jeffrey Braithwaite, Russell Mannion, Holger Pfaff, Siri Wiig

**Affiliations:** 1 Australian Institute of Health Innovation, Macquarie University, North Ryde, NSW, Australia; 2 Health Services Management Centre, University of Birmingham, Birmingham, United Kingdom; 3 Institute for Medical Sociology, Health Services Research and Rehabilitation Science (IMVR), University of Cologne, Cologne, Germany; 4 SHARE-Center for Resilience in Healthcare at the Faculty of Health Sciences, University of Stavanger, Stavanger, Norway

**Keywords:** change, complexity, connections, context, cultures, improvement, improvement science

## Abstract

**Background:**

Significant and sustained change in healthcare is often challenging. One explanation is the need to satisfy many stakeholders who may have different interests. In the context of the Special Issue on improvement science, multiple and aligned changes at different levels are often needed to create genuine change.

**Objective:**

To propose ideas about why these types of change are hard to achieve, and present a model—a heuristic device—for guiding such change by those responsible for improvement in healthcare.

**Methods:**

Narrative review and conceptual synthesis. The author team combined (i) long-standing expertise in health system improvement and implementation science with (ii) targeted identification of relevant literature across complexity, organisational culture, context, and relational/coordination scholarship. We iteratively coded and clustered concepts, compared them with established improvement and implementation frameworks, and refined the model through consensus via a range of meetings and discussions of healthcare improvement over a ten-year period.

**Results:**

The synthesis yields the Four Cs model—Complexity, Cultures, Contexts, and Connections—as a coherent way to diagnose and design improvement efforts. The model highlights the need for continual coordination across levels as contexts shift, and it reframes ‘change agents’ as ‘improvement architects’ who work deliberately with system dynamics, cultural patterns, contextual constraints, and relational coordination.

**Conclusions:**

The Four Cs model integrates four well-established constructs into an improvement science-oriented heuristic intended to support reflection, sensemaking, and design. We propose it as a practical guide and agenda for empirical testing, refinement, and debate within the improvement community.

## Background

In the context of the Special Issue in *Frontiers in Health Services* on ‘Frontiers of Improvement Science in Health Services’, we present a perspective on change with qualified recommendations for healthcare leaders responsible for healthcare improvements. We want to say at the outset that enhancement of delivery systems, from policy to practice, is a formidable, wicked problem (1, 2). Reed and colleagues (3) specify four distinguishing features of improvement science in their leader in *Frontiers*. They seek to conceptualise the field in terms of *complex social systems*; note that improvement science scholars take a *holistic, systems view* in contrast to disciplines which privilege controlled studies; observe that the community focuses on the *practical application of knowledge*; and see the field as one which *engages pluralistically with a wide-range of stakeholders*.

We respond to those features and the invitation to contribute this way. We agree that healthcare services are *complex*. We also argue that the ecosystems that provide care comprise highly *contextualised* settings with differing, multi-faceted *cultures*. It is also defensible to say that *connections*—the relationships that underpin care—are vital. By describing the system that way, we mean that healthcare is a multiplicity of social processes that are interactive, distributed, dynamic, relational, socially embedded and nonlinear. Intricate healthcare systems and social structures evolve and adapt over time, and are negotiated orders in the classic theoretical paradigm of Anselm Strauss (4, 5).

### Overarching aim and problem statement

Here, we want to deal with four aspects of the healthcare system that Reed et al., challenged the community about (3), and that we opened with: complexity, culture, context and connectivity. We have labelled this the *Four Cs model*. We believe, after decades of researching healthcare between the authors, that these are the fundamental social structural elements—the underlying systems DNA—of healthcare, and are essential to an understanding of both improvement, and improvement science. We want to analyse that Four Cs claim, and examine if it addresses a longstanding problem: to move from outdated models of mere change with “change agents” at its core, to an “improvement architect” model.

By change agents, we mean individuals or groups who seek to promote or implement change initiatives within healthcare organisations or systems. And by improvement architects, we mean those who work more systemically to design, align, and continually adapt the conditions under which improvement can occur, taking account of complexity, cultures, contexts, and connections. In combining the four constructs in one model, we aim to go beyond existing complexity and implementation frameworks. That said, we note in passing earlier work, by way of example, which has looked at context, connection and complexity in doctoral programs (6) and other work on transforming cultures in an integrated framework (7). Box 1 provides our definitions.

BOX 1 **Terms and concepts used**

**Glossary of terms**

Adaptation The capacity to adjust to internal and external circumstances; usually thought of in terms of modifying behaviours over time

Adaptive capacity The ability and competencies within a system to cope and adjust to variability, change, threats and opportunities

Agents The individual components of a complex system—typically, individuals, whose capacity for sense-making means they can learn and adapt their behaviours across time

Change agents In this context, change agents are individuals or groups who seek to introduce, promote, or implement change in healthcare organisations or systems. They are typically understood as people who champion reforms, encourage adoption of new practices, mobilise others, and help move organisations from a current to a desired state. The term often implies a relatively bounded role focused on driving or facilitating change initiatives.

Complex Adaptive System A dynamic, self-similar collectivity of interacting, adaptive agents and their artefacts

Complexity The behaviour embedded in highly composite systems or models of systems with large numbers of interacting components (e.g., agents, artefacts and groups); their ongoing, repeated interactions create local rules and rich, collective behaviours

Connections Relationships, links and networks between people, teams, organisational units or whole enterprises involving exchange of ideas, power, resources and perspectives

Context The conditions, circumstances, situations and environment in an ecosystem of care that differ from other conditions, circumstances and situations in other ecosystems

Core of care The foundational nexus of a health system—the point at which clinicians and the patient, client or consumer meet for services, treatment, education, rehabilitation, interventions or palliation

Culture The sum of the shared values, attitudes, and beliefs across part of or the whole of an organisation (e.g., across the division of medicine, or an entire hospital or health service)

Emergence Behaviours, structures, patterns or properties that are built from smaller or simpler entities, the characteristics or properties of which arise through the interactions of those smaller or simpler entities; the larger entities are one level up in scale, and manifest as social structures, patterns, or properties

Feedback loop A recursive mechanism creating reciprocal behaviours that reverberate back in on themselves; a positive (self-reinforcing) feedback loop increases the rate of change of a factor, creating more of its own output; in a negative (self-correcting) feedback loop, the output responses dampen the change or modulate its direction

Implementation science The science and social science of the processes of translating research into practice, understanding what influences translational outcomes, and evaluating the adoption of interventions

Improvement architects In this context, improvement architects are those responsible for designing, coordinating, and adapting improvement efforts across the multiple levels of healthcare systems. Unlike change agents, who are often conceived as promoting discrete initiatives, improvement architects work more systemically: they attend to complexity, cultures, contexts, and connections; align stakeholders; shape the conditions for change; and continually redesign improvement efforts as circumstances evolve. The term emphasises not simply advocating for change, but deliberately constructing and stewarding the social, organisational, and relational infrastructure through which sustained improvement becomes possible.

Macro, meso, micro levels Hierarchical levels of the health system, from high level (macro, e.g., the whole system or society, regulation, national bodies) through mid-tier (meso, e.g., organisations delivering care) to the front lines of the system (micro, e.g., clinical teams, wards, operating theatres)

Negotiated order The idea that everyday organisational life is shaped not just by formal rules, but by ongoing negotiation and adjustment among the people involved

Network An interlocking web of relationships or connections at varying levels of scale in a system; the agents or artefacts are the nodes and the relationships between them are lines or vectors, which together describe the structure of the interactions of the network's membership

Path dependence Current events and circumstances are influenced, and can be determined, by prior events and circumstances, harking back to the origins of the entity or system; path dependence underpins the point that ‘history matters’

Perturbation An internal or external disruption or unexpected event that affects normal patterned behaviours, structures or processes; often thought of as an external disturbance or interruption to the current state-of-affairs

Self-organisation The way in which agents interact to coordinate their own circumstances, workplaces, processes and procedures, such that they order their work and they autonomously, or semi-autonomously, organize their localized behaviour; this can occur passively or actively

Sensemaking Methods by which individuals figure out what is going on around them; a typically social process among agents in which they come to a shared meaning of their experience, and is necessary for action in the face of ambiguity or uncertainty

Social network A set of people who have relationships, communications, ties, or interactions that connect them

System dynamics An analytical modelling methodology used for problem solving, which combines qualitative and quantitative data and identifies the fundamental elements of a system, and how they influence one another over time

Tipping point A critical point in a system in which a kind of radical, potentially irreversible, change may occur, resulting in a different state of system behaviour, which can settle into a new equilibrium

Wicked problem A problem that is hard to define clearly, has many causes, involves conflicting perspectives, and cannot be solved once and for all.

## Methods

### Analytical approach and design

Narrative review and conceptual synthesis (theory-building).

### Team expertise

The authors draw on complementary expertise in health system improvement and implementation science, organisational culture, health services research and resilience.

### Literature identification

We combined author libraries and seminal texts with broad-based searches using terms such as ‘complex adaptive systems’ AND healthcare, ‘organisational culture’ AND quality improvement, ‘context’ AND implementation, and ‘relational coordination/connections’ AND healthcare improvement.

### Inclusion focus

Peer-reviewed empirical studies, major syntheses and foundational theory relevant to improvement and implementation in healthcare; we privileged sources that inform practical design and execution.

### Synthesis steps

Independent compilation of candidate constructs; clustering of concepts; iterative refinement to four constructs based on explanatory power and usefulness for improvement design; and collaborative sense-checking against counterexamples and alternative frameworks. We iteratively developed concepts, compared them with established improvement and implementation frameworks, and refined the model through consensus via a range of meetings and discussions of healthcare improvement over a ten year period.

### Reflexivity/positionality

The authors are active contributors to the improvement and implementation science literature; to mitigate confirmation bias, we deliberately sought disconfirming literature and compared our synthesis with established frameworks (see Discussion).

Sources: Definitions are synthesised from the literature in each section below, especially Reed et al. (3), Braithwaite et al. (8), Braithwaite et al. (9), Davies et al. (10), Scott et al. (11), Ommen et al. (12), Wiig and Fahlbruch (13).

## Results

### Our derived perspectives on the dynamics of care

At an overarching level, health systems follow an analogous pattern, regardless of country, structure or funding models. They differ in many details across region, setting or provider, but they are more similar than different. Clinicians provide front-line care to many different types of patients with a multitude of conditions. Patients seek care episodically depending on need, diagnosis and preference. Beyond clinicians providing direct care, there are many other personnel who influence, fund or support front-line activities; for example, policymakers, commissioners, regulators, managers, scientists, pathologists and x-ray personnel, administrative staff, porters, caterers and cleaners. Large proportions of a nation's financial resources are devoted to care (the OECD average is around 11% although the US's *per capita* expenditure is much higher), and the staff involved deploy many kinds of technology such as computers, beds, stethoscopes, diagnostic imaging machines, operating tables, scanners, drugs and tests. Staff are provided with, co-create, adhere to, workaround and sometimes reject or ignore many rules, regulations, policies and guidelines meant to shape the delivery of care. By any measure, then, delivery systems are *complex*.

Sociologically-speaking, there are different cultural groupings operating at differing levels of healthcare, with distinguishable levels of power, influence and resources. Each caring organisation has a distinctive, patterned *culture.* There are various other sub-cultures—professional sub-cultures differentiating medicine, nursing and other professionalised tribes, for instance, and sub-cultures of policymakers, leaders and other groupings.

Each organisational setting can also characterised *contextually*, which is a related but distinguishable construct to that of culture. Context can be understood as the environment which encapsulates the content of care, and is the environmental ecosystem.

This comes together in a melange of interactions, groups and social structures focused on treatment and care. One key to understanding the complexity, culture and context lies with relationships. This is the *connections* between the agents, groups and components of the system, which are resonant and dynamic.

In the constant stream of caring, policy and practice activities, problems are encountered by healthcare's agents (a policy does not quite fit the circumstances, a government body introduces a legislative change, a series of patient deaths triggers a national enquiry, a patient condition unexpectedly worsens, a computer system goes down, some staff call in sick, a patient receives the wrong drug dose and the like). On the front lines, adaptations and adjustments occur all the time to behaviours, clinical pathways and guidelines. People use tools, their expertise, the advice of others, initiatives, hierarchies and workarounds to achieve their care goals. Many minor alterations, nudges and fine-grained modifications to the system are made over time. We want to address all of these in the Four Cs model (Box 2).

BOX 2 **The Four Cs model—selected features**

**Complexity** analyses place considerable attention on the non-linear dynamics of the system. For example, *emergence* expresses the behaviours that occur at the next level up; agents create higher-order, patterned social structures arising from their relationships: collaborating individuals gather in groups, for instance, and multiple groups assemble into organisational divisions. *Unpredictability* suggests that there are always unexpected occurrences. *Path dependence* says that current events are influenced and to some extent determined by prior events and circumstances back to the system's origins.

Alongside these complexities and contingencies**, culture** focuses on shared, *taken for granted behaviours, values and attitudes* expressed in everyday organisational activities. It is often described as ‘*the way we do things round here*’ which is distinguishable from ‘*the way they do things over there*’.

**Context**, our next construct, draws to attention *the overarching environmental setting and surrounds*. It is mostly understood with reference to social and psychological variables, e.g., hierarchy, gender, social identity, and the influence of the physical settings and the *local situations* in which people operate.

**Connections** highlight *the inter-relationships* between the agents, groups, organisations and institutions directly delivering or supporting the delivery of care. Connections are centrally about *interactions* amongst the many stakeholder groups which create or co-create care.

The following statements are synthesised from the literature cited in each subsection and our own expertise in the field, and are offered as a practical summary of the Four Cs model's implications for improvement design and execution. The synthesis yields the Four Cs model – Complexity, Cultures, Contexts, and Connections—as a coherent way to diagnose and design improvement efforts. The model highlights the need for continual coordination across levels as contexts shift, and it reframes ‘change agents’ as ‘improvement architects’ who work deliberately with system dynamics, cultural patterns, contextual constraints, and relational coordination.

[Note: References supporting this Box 2 summary are cited below]

### Accounting for the Four Cs model

We propose the Four Cs model as a way to tackle this thorny problem: that much of what stakeholders in the system attempted to enact in terms of improved healthcare is not improvement but relatively superficial change—and sometimes, misguided change, or pseudo-change, or mere change for change’s sake, or change with good intentions but poor execution, or change with unintended or dysfunctional consequences.

Because of poor change practices, healthcare has not made sufficient progress in providing better arrangements to enhance care to patients in a scientific way. Reasons tendered to explain the lack of progress include that healthcare is conservative and resistant to change; and that meaningful improvement (e.g., changing clinical behaviour, or measurably enhancing patient outcomes) is relatively difficult yet making surface-level change (e.g., reorganising providers into new groupings, substituting an existing IT system for a more advanced version, or altering a role description) is relatively easy. Additionally, the vastness of the caring enterprise frustrates across-the-board initiatives or those which seek to scale and disseminate new practices, procedures or ways of working.

To understand change more deeply, and to have a better model for improvement, we need to factor in the essential elements in our Four Cs model: how people deal with complexity, culture, context and connecting the people in ways that support services to improve rather than to superficially or incrementally change things. We conceptualise these essentials in Box 3.

BOX 3 **The elements of the Four Cs model**

Shifting from mere change to improvement requires a deeper understanding of the individual expressions and sum total of complexity, culture, context and connectivity. Let OI = Organisational Improvement; ME = Management effectiveness or engagement; CL = Complexity; CU = Culture; CX = Context; and CN = Connections.

We express this in a formula:

OI=ME⋅(CL+CU+CX+CN)

And refined further, weighting (*w*) the four elements depending upon needs, to give precedence of one over others where necessary, we have a functional definition:

$$\mathrm{OI}\;=\;\mathrm{ME}\;\cdot \;(w_{1}\mathrm{CL}\;+\;w_{2}\mathrm{CU}\;+\;w_{3}\mathrm{CX}\;+\;w_{4}\mathrm{CN})$$

Note that this ‘equation’ is a heuristic device: a compact way to communicate that management engagement amplifies attention to each of the Four Cs. It is not a literal mathematical model intended for measurement or prediction.

That said, we turn to an analysis of each of the four constructs in our argument. We deal with each of complexity, culture, context and connectivity in turn, and then provide examples of the kinds of systems improvement we hope to see, applying this model. We want to shift people such as those trying to create a more evidence-based world of practice from thinking of themselves as *change agents* to *improvement architects* via the Four Cs model.

## Discussion

### Complexity and healthcare improvement

There are many ways to understand complexity, but we see two as important. The first is to contrast linear depictions of how health systems function with complexity depictions. The second is to briefly examine how our understanding of the health system got to the present so we can deploy complexity thinking as a useful tool to make future improvements.

#### Linearity versus dynamics

**Figure 1:**
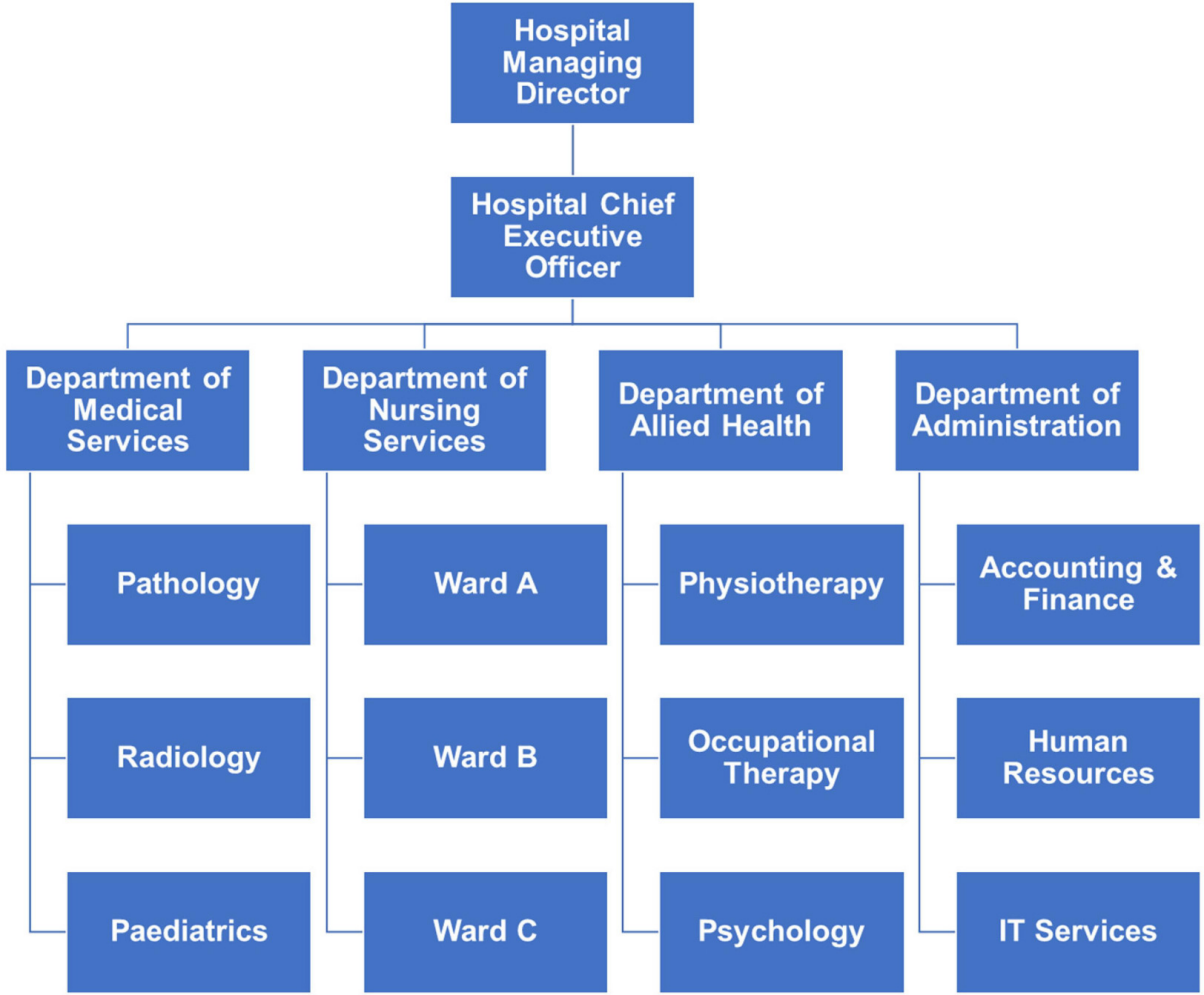
Example of healthcare organisation chart. Source: Authors’ conceptualisation, sysnthesised from many representations.

Change agents often initially describe the system they seek to change, and all-too-often visually portray this in a simplistic way; as line-box-and-arrow depictions. Graphic presentations of this non-dynamic way healthcare “works” are common (see, for example, Morell-Santandreu et al. (14). A typical example is the organisational chart (Figure 1), presenting a two-dimensional snapshot, representing the way personnel are organised hierarchically, who reports to whom, and how responsibility and reporting lines operate.

**Figure 2:**
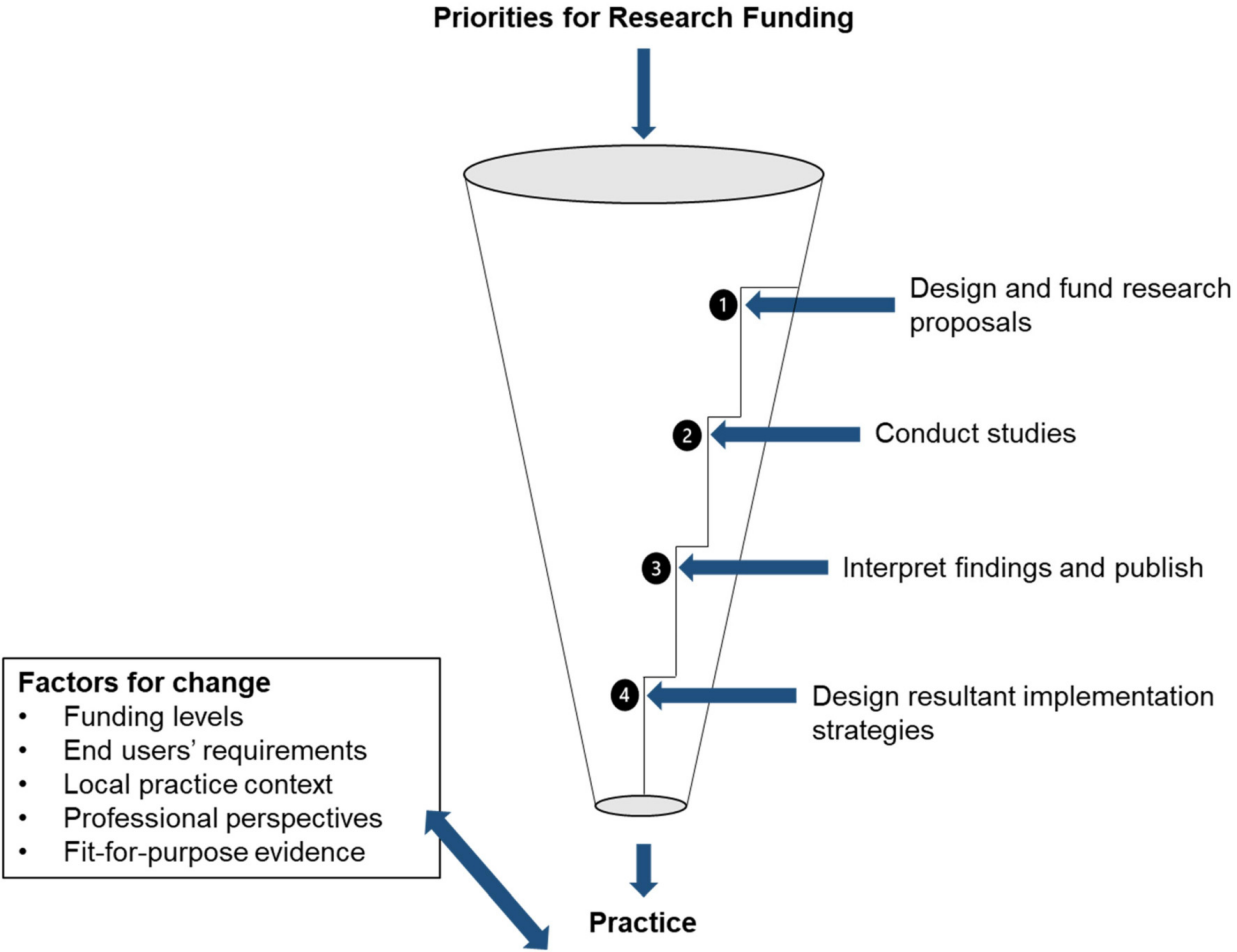
Exemplar of the “pipeline” concept of doing and disseminating research to promote evidence-based practice. Source: Author's conceptualisation, sysnthesised from many projects.

Others have schematically conceptualised, again in a relatively step-by-step way, the way policy, research findings or other evidence find their way into clinical practice (see, for example, Green (15)). Figure 2 is an example.

Such depictions suggest that reporting lines (Figure 1) and evidence-to-practice processes (Figure 2) are certain and predictable. People purportedly know where they are in the hierarchy (Figure 1) and which events happen before and after others (Figure 2). Shown this way, it seems logical and obvious that the boxes on the chart, steps in the process or ordering of people or events can be understood, and then altered to suit different purposes. Making the system more streamlined, for example, or adding or taking away accountabilities, funding or tasks are easy fixes. Alter the lines, boxes and arrows on the organisational chart in Figure 1, and then correspondingly alter the arrangements *in situ*, in the real world of the aged care facility or general practice and change occurs*.* Shift the research priorities, what gets funded, and how well the implementation strategies are designed and taken up, and practice becomes more evidence based as exemplified in Figure 2.

The real world is much more messy, imprecise, uncertain and unpredictable than these depictions suggest. Linear, mechanistic and simplified versions of how healthcare works such as these are incomplete, and must give way to more complex accounts. That deserves a little historical reflection.

**Figure 3:**
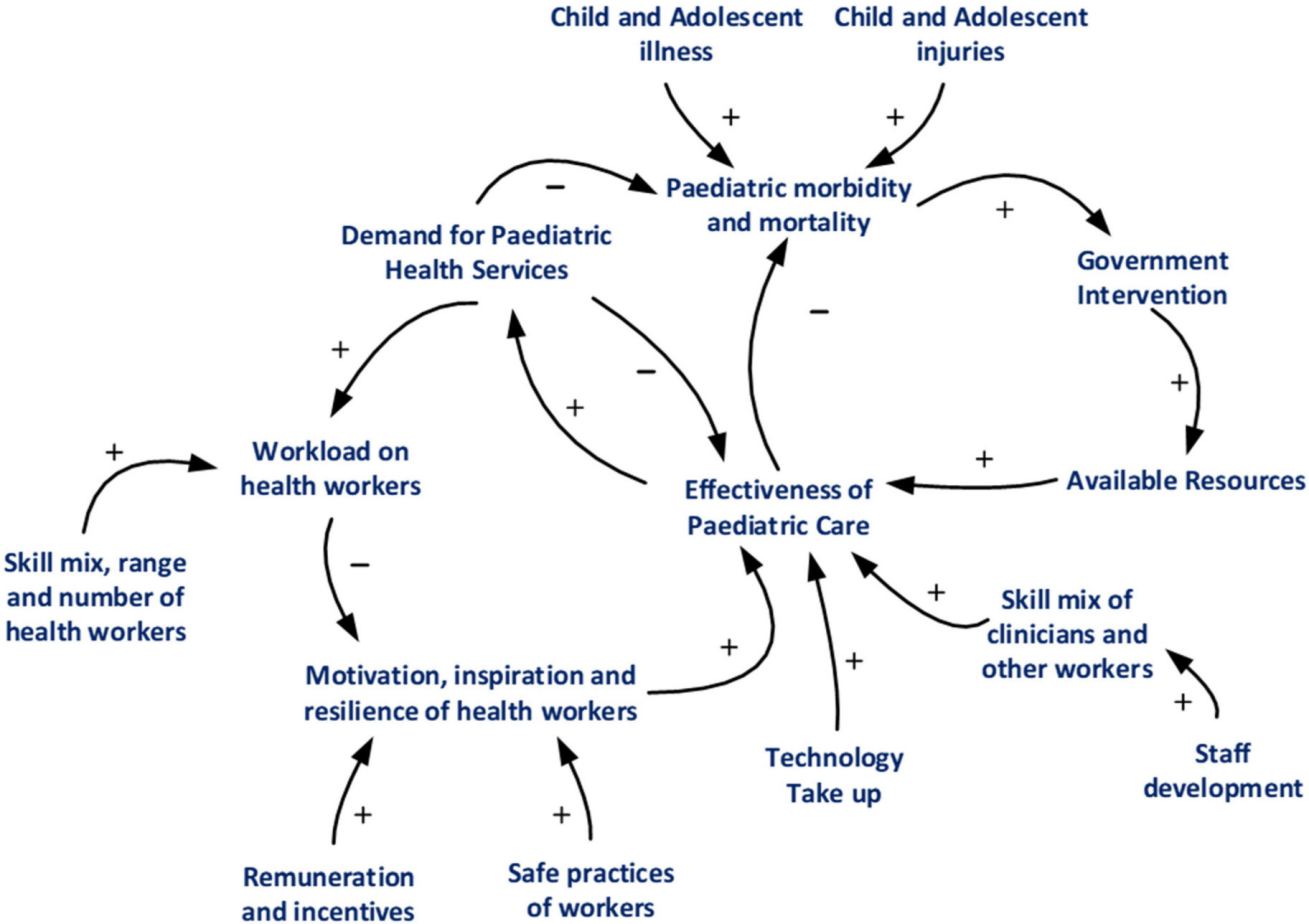
Partial causal loop diagram for paediatric care. Source: Authors’ conceptualisation.

Before the rise of systems thinking, it is likely that many people subscribed to the normative, instrumental and simplified views of social structures depicted in Figures 1, 2. The earliest social theorists of systems such as Von Bertalanffy (16), Checkland and Scholes (17) and Van de Ven et al. (18) began to alter such perspectives. That new paradigm was more sophisticated than mechanistic descriptions of organisational function but was not yet infused with complexity thinking (19). That came later, by many contributors, including, in healthcare, Bar-Yam (20), Bar-Yam et al. (21), Braithwaite et al. (22), Braithwaite et al. (8), Checkland and Scholes (17), Hawe et al. (23), Hawe et al. (24), Leykum et al. (25), May et al. (26) and McDaniel and Driebe (27). By drawing on scholarship as far afield as biology, evolution, the sociology of organisations, policy studies and physics, richer descriptions of systems began to emerge. In this newer frame, healthcare settings, organisations and institutions are understood to be Complex Adaptive Systems (CASs) (8, 28, 29). To illustrate, Figure 3 shows part of a causal loop diagram of clinical services, where the arrows depict the interactions and relationships between the components of the system: for a further example, see Sooka and Rwashana-Semwanga (30).

**Figure 4:**
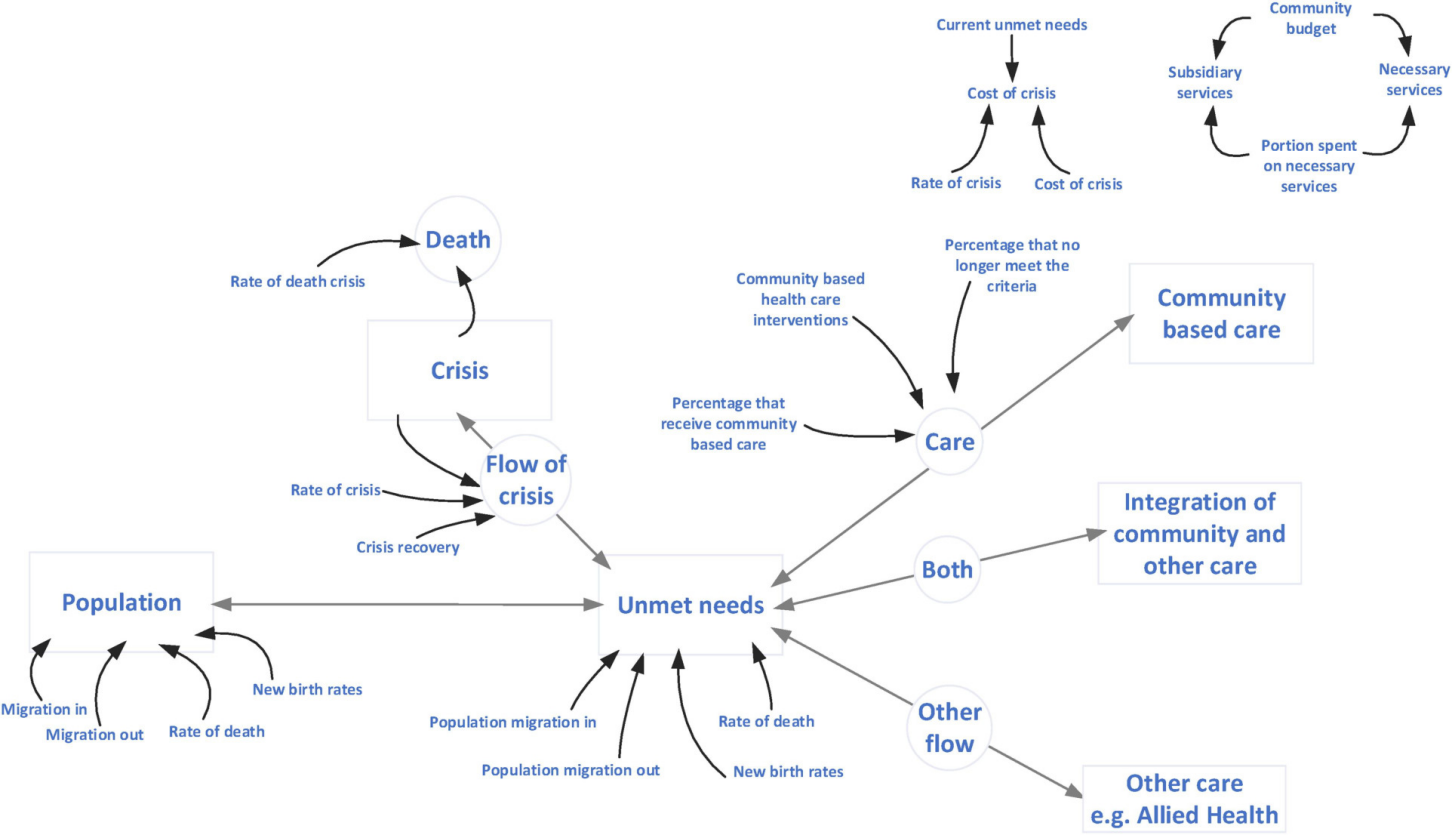
Partial representation of a systems dynamics model for community-based care. Source: Authors’ conceptualisation.

Figure 4 provides, again to illustrate, a systems dynamic model of the processes by which, in a social care system, work, relationships, connections and behaviours shift across time. For a further example, see Brailsford et al. (31).

Figures 3, 4 draw attention, then, to the *dynamic* nature of caring systems. The causal loop diagram (Figure 3) and systems dynamic model (Figure 4) represent the iterative connections, informal encounters, self-organisation, emergent and oftentimes messy interactions between clinicians, patients, support personnel, equipment and buildings housing the agents.

#### Summary of our rendering of complexity

Linear diagrams such as in Figures 1, 2 portray healthcare as a streamlined, idealised model, and complexity renderings as in Figures 3, 4 expose nonlinear dynamics, multi-variability, richly-constituted interactivity, and adaption over time. These latter kinds of accounts are more effective as representations of the real-world than the more simplistic, structured portrayals. We maintain that linear depictions can lead to change, but rarely to improvement. They often lead to restructuring the organisation chart, which alters the boxes and labels on the organigram, but often, little else. Dynamic models suggest that quick fixes are hard to accomplish, but if the system is altered—influenced, perturbed, or provided with negative or positive feedback—it might lead to more deep-seated improvement rather than mere change. Complexity models can help pinpoint to where agency is exercised and where self-correcting feedback loops can be accentuated.

### Culture and healthcare improvement

Moving to the second Four Cs element, healthcare reforms frequently invoke notions of culture change, seemingly as a means of enhancing health care. Implicit in this thinking is the notion that creating the right kind of culture will facilitate better functioning workplaces and higher-quality care. A question is whether such talk is vacuous rhetoric, or whether this framing of healthcare organisations and systems in cultural terms offers useful insights that might help to drive improvements in care.

How are cultures linked to change and improvement? Can cultures in healthcare organisations be purposefully shaped and managed to beneficial effect? (32). Notwithstanding its widespread use the concept of organisational culture is far from being universally agreed upon (33, 34). Instead, organisational culture is contested, with many definitions and elaborations (35). But central to many definitions is the assumption that culture comprises that which is shared and taken for granted between organisational members. This might include the values, beliefs and assumptions which underpin everyday organisational life, as well as the ceremonies, symbols, rewards and taboos which guide common and repeated patterns of behaviour.

Traditionally, approaches to understanding organisational culture can be divided into two camps: “corporate culturist” and “interpretive” approaches (36). The *corporate culturist* approach views culture as an *attribute*—something that an organisation *has*. Here, culture is conceived as an operational variable which can be managed and manipulated by managers alongside other variables such as strategy and structure. Culture change therefore is directed at ‘reengineering’ an organisation's value system. Much popular management literature, airport bookshop bestsellers and policy rhetoric adopt this approach. In contrast, “*interpretivist”* approaches construe culture—as something that an organisation *is*. Cast in this way, culture arises spontaneously from everyday social interactions and is actively lived and shaped by organisational members as they interact. In this approach, taking a steer from Habermass, culture is reproduced constantly (37). While managers might be able to modify some outward manifestations of culture, the basic assumptions held by organisational members may not fundamentally change. The attention therefore shifts from concerns about what organisations do and how they can do this more efficiently, to how organisation is accomplished and what it means to be organised.

Despite disputes over the precise definitions of organisational culture, most commentators agree that it is layered. One of the most commonly used presentations of cultural layering was proposed by Schein (38) who identified three levels of cultural analysis of ascending importance (Box 4). In this schema, the observable patterns of behaviour are explicitly linked to deeper levels of shared cognitions and (harder to access) assumptions, unconscious beliefs and precognition biases.

BOX 4 **Schein’s tripartite model of culture**

- Artefacts: are the surface-level attributes that can be immediately seen, felt, and heard when one enters an organisation. In a healthcare facility, they might include presenting, observable behaviours; and the physical and social environment, including the layout of work spaces, the spatial location of wards, staff rotas, job titles, and dress codes.
- Beliefs and values: are the largely unwritten informal rules used to justify particular behaviours; they provide a rationale for choosing between alternate courses of action, and distinguish organisationally-sanctioned conventions or perspectives that are judged “right” or “wrong”.
- Basic assumptions: deeper still, but much harder to access and observe are the often unconscious and unexamined expectations, perceptions, and presuppositions shared by organisational members that underpin day-to-day work.

Those familiar with healthcare organisations will recognise that their cultures are usually far from uniform or coherent. Some cultural attributes may be visible across an organisation, but others may be prominent only in particular sub-cultures. Different sub-cultures may emerge, for example, across different occupational or professional groups, or between teams physically separated. Conflicts between cultural subgroups may reflect differences in power, legitimacy or outlook, and they can be as much about a struggle for expression of identity, meaning, and purpose as they are competition for resources, autonomy, and control (32).

Doctors and managers differ on many cultural dimensions. This can lead to misunderstandings and tensions in working relationships and approaches to improving patient care. For instance, healthcare professionals and managers may use the same terminology when speaking of patient care (relating to quality, safety, or the use of evidence, for example) but mean different things and understand the “proper” solutions in different ways. Thus, deep-seated resistance by powerful clinical groupings to management-instigated changes (such as structural reorganisations and mergers) can help to explain the failure of such initiatives to take root. This can happen in reverse. Clinicians may advocate for a modification, say in an IT system that doesn't work for them, or the way their hospital investigation of a clinical error is being run, and management might be resistant to the clinician-led change proposal.

Moreover, it can be difficult to decide whether counter-cultures are stubborn resisters to necessary change, simply defenders of their own traditions, or perceptive opponents of damaging new directions. Understanding this is essential for any effective cultural diagnosis before embarking on health improvement. Culture may also be a prescription for improvement when faced with a toxic culture. And organisational culture itself is a sub-culture within a larger set of supra-cultures, including the health system-wide culture, or indeed, national culture.

A systematic review of 62 studies found a “consistently positive association between culture and outcomes across multiple studies, settings, and countries” (39). So: if there is a relatively productive, positive culture in a healthcare organisation, it is more likely that patient outcomes will be better than in those organisations in which there is a negative, or non-productive culture.

#### Summary of our rendering of culture

As with our discussion of complexity, linkages between culture and health improvement are likely to be highly contingent, and nonlinear, making it an inherently difficult domain to study, and hard to draw generalisable recommendations applying across diverse contexts (40). Thus, the relationship between culture and care improvement is context-dependent. One key difficulty is that although most attention has centred on how culture may affect performance, it is plausible that certain cultures arise from high-performing organisations. More likely still is that culture and performance improvement are mutually constituted and reinforcing, and dependent on wider contexts and influences (33, 34). Indeed, the widely used phrase “the way things are done around here” could be interpreted as much a definition of *performance* as it is of *culture*. Current policy prescriptions to improve healthcare performance through cultural transformation are therefore in need of more secure evidence base, underpinned by a more sophisticated understanding of cultural dynamics, including the role of sub-cultures in supporting or undermining necessary health care reform and reconfiguration.

### Context and healthcare improvement

Improving healthcare requires us not just to apprehend the complexity and culture, but the organisation's or system's strategy, ethics, values, knowledge, politics and power. Strategy, ethics and the values of organisational citizens are needed “to do the right things”. Knowledge is required “to do the right things right”. Politics and power are essential to implement and integrate activities. This brings us to organisational context (41), our third element in the Four Cs model.

**Figure 5:**
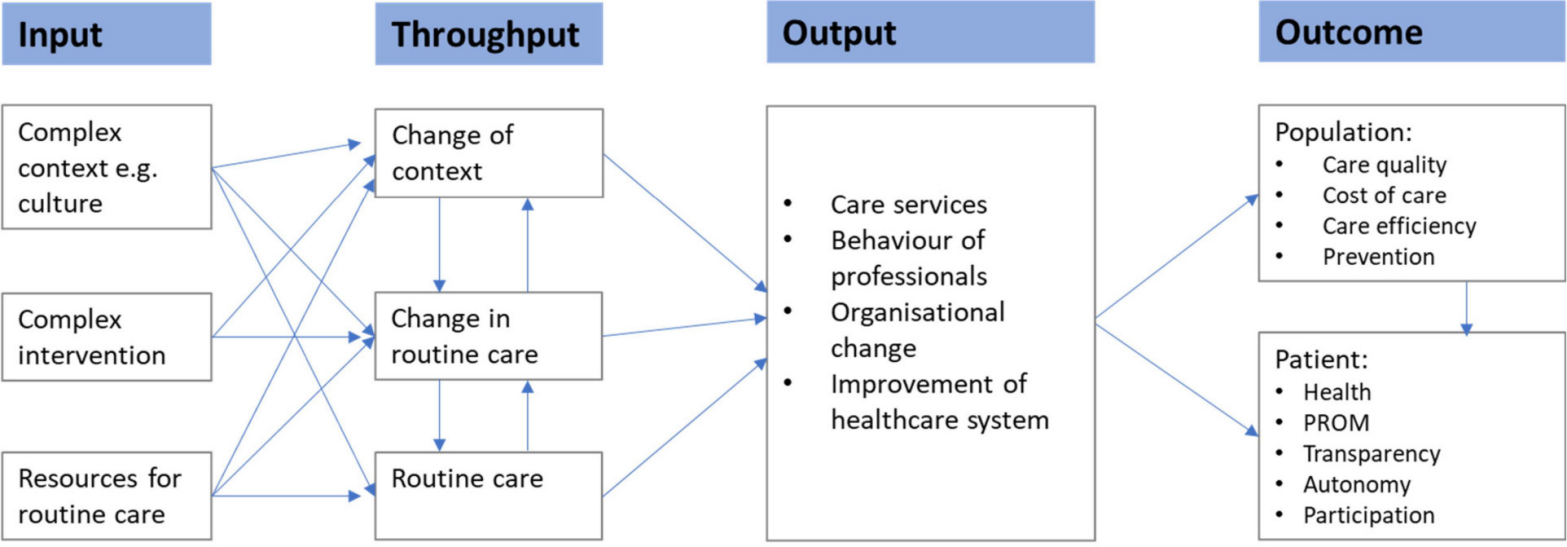
A modified process of care model. Source: Authors’ conceptualisation.

Context is a relative construct—it differs across environments and workplaces—because it relates to content as a reference point. It can be defined as the environment of a specific content, and radiates out from the core of care. There is always contextual variation—distinguishable features in ecosystems lead to things being done differently, even though at the core of care there is a self-similarity. What is similar is the familiar clinicians applying technologies and human ingenuity to provide care to patients, and patients applying their knowledge and expertise to the decisions about their care. Going further, at the core of care is the application of health technologies to enhance or preserve health and/or quality of life through prevention, diagnosis, therapy, nursing care, rehabilitation and/or palliation (42). The core of care, the target of evidence-based medicine, consists of an “input-throughput-output-outcome” chain (Figure 5; and see Schrappe and Pfaff (43).

The input consists of the resources necessary to provide high quality routine care as well as patients’ understanding of their condition, willingness to get better, and motivation to contribute. Healthcare organisations, then, consist of care processes in their deeply-embedded contexts (care structures, non-care structures, processes and cultures). The outputs of the healthcare system are patient-related services with outcomes that have an effect on both individual and population levels. A disease episode is often characterized by a string of different “input-throughput-output” links, resulting in a long care chain (e.g., emergency-acute/clinic-rehabilitation/clinic-general practitioner) with different feedback loops.

The context of care on this analysis consists of all the environmental factors surrounding the content of care with the core of care in the centre. It can be categorized along two dimensions. The first comprises psychological systems, social systems, machines, and organisms (44), as well as the physico-chemical and cultural systems (45, 46). The second dimension relates to three levels of context: the micro-, meso- and macro-contexts (47, 48). The micro context is the level of social interaction (e.g., physician-patient interaction, or professional-professional interaction). The meso context consists of organisations and networks of organisation-clusters of hospitals, general practices or aged care facilities. The macro context is at the level of populations, societies or government (47).

Autopoietic systems like humans or front line teams (49, 50) are fundamental to context (51). With the context at the core, physicians adapt their therapy strategy to the personality and preferences of the patient. Ideally the patient co-produces their care during shared decision making. The micro level is often construed as the microsystem. The level above the micro is the meso level—as we define it here, the whole organisation. The organisational level is able to influence the micro level by establishing the sociological rules of behaviour. At the meso level, the healthcare system, population, society or government influences the meso-context organisation, for example, via mandated policies or legal regulations.

#### Summary of our rendering of context

All interventions or activities within the system are context-sensitive. There are different types of possible context effects: confounding, mediating, moderating, moderated mediation and interference (52–57). These types of influence have to be considered when designing evaluations, conducting interventions and experiments, and implementing and integrating localised healthcare improvement.

### Connections and healthcare improvement

We conceptualise connections, our final Four C element, as those relationships that keep the parts of the system together—the mechanisms of interpersonal, inter-agent and inter-organisational associations. Connections, conceptually, describe the entangled inter-dependencies between the agents, groups, organisations, and institutions that deliver, or support the delivery of, care. Connections enable the formal structures and processes that are visible on organizational charts and in role description documents to function in the real world. They are the coordination that is required for healthcare services to carry out their care mission (58). Connections also integrate the informal, tacit knowledge and power needed to enable healthcare organisations to pull in the same direction.

The crucial role of connections makes what seems impossible—to bring together all the multitude of otherwise fragmented, disparate people and their affordances to produce good care—possible. On its face, it seems highly infeasible that complex health systems, across different contextual settings, with cultural differences, siloed structures, divisions of responsibility, politics, and contextual differences, can work at all, let alone effectively. However, care happens every day. Thus, to improve services and deliver quality care is to pay attention to and understand the crucial role of connections and make sure people have time, resources, and interest in optimising their relationships across the system (59, 60).

In studies of leading hospitals (e.g., Bate et al. (61)), researchers identified multiple connections. Bate et al. (61) for example, showed how leaders needed to work on power dynamics, emotions, and relational issues to succeed in improving hospital service quality. These are elements often neglected in improvement projects. To give another example, the Bate et al. (59) findings were echoed in a recent intervention study implementing a quality leadership guide in Norwegian nursing homes and homecare services. Here, leaders who enabled better work processes involved personnel, and were interested in establishing sound working relationships and conditions, factoring in the complexity of their environment. They also ensured continuity of care and presided over a clear social structure, and empowered professionals, connecting people with different competencies to support each other (62–64). Johannessen and colleagues’ (64) study also found that leaders needed to use their skills and contextualised knowledge, encouraging staff to have large degrees of freedom for manoeuvring within their complex organisation.

These key dimensions of connections illustrate how people in care settings can use their professional and interpersonal skills to make links to each other and exercise their competencies. The goal is to shape a culture of inclusion where people are more likely to support each other.

#### Summary of our rendering of connections

Raising the importance of connections, recent work on system-, organisational- and group-factors related to adaptive capacity—the ability of people to flex and manoeuvre—in hospital teams, underscore the point. For example, in a recent case study among hospital teams, leaders who were able to collect and connect information on formal processes and understand personal and team preferences were able to support adaptive capacity. Leaders using their experience and mobilising relationships enabled teams to go the extra mile for patients and deliver quality care despite shortages and time pressures (65, 66). Focusing on connections made the culture and context come together in these complex settings.

### Bringing the constructs together: the Four Cs model in action

We claim that paying attention to complexity, culture, context and connections is more likely than other options such as altering the arrangements in Figures 1, 2 to create the opportunity for fundamental improvement rather than superficial change. At present, most organisations, while well-intentioned and well-meaning, end up fiddling at the edges. Common transition programs typically involve funding a project for a time, and then aiming to “bed it down”. Then, everyone moves on to the next initiative. Change in healthcare is therefore, for the most part, short term and discontinuous. We know that most change projects are judged as failures or fall short of aims, expectations or hopes for them (67–69).

So, what needs to be done, to do change differently? To get closer to a better vision of improvement? Let's use our Four Cs formulae, and provide examples.

**Complexity** $\mathrm{OI}\;=\;\mathrm{ME}\;\cdot \;(w_{1}CL\;+\;w_{2}\mathrm{CU}\;+\;w_{3}\mathrm{CX}\;+\;w_{4}\mathrm{CN})$

On the basis of the foregoing, linear change models are insufficient. Strategies and tactics to facilitate complexity include:

- Make progress in understanding the perspective of multiple stakeholders; encourage a willingness to undertake negotiation and trade-offs between competing priorities, recognising non-linearity
- Using feedback loops such as Formative Evaluation Feedback Loops (FEFLs) (2) to stimulate improvement
- Changing the narrative around path dependence such that the story of the organisational culture shifts to a more positive version
- Using system thinking, and systems models, such as causal loop diagram and system dynamics models, to more deeply understand variables that focus attention.

**Culture** $\mathrm{OI}\;=\;\mathrm{ME}\;\cdot \;(w_{1}\mathrm{CL}\;+\;w_{2}CU\;+\;w_{3}\mathrm{CX}\;+\;w_{4}\mathrm{CN})$

However we define it, culture is a deep-seated organisational attribute. To the extent that it can enable or impede change, it must be factored into any improvement approach or implementation exercise. Strategies and tactics include:

- Working with Schein's or a similar model so that, to the extent that someone can influence culture, they attend to adjustments to the artefacts, beliefs and values, and basic assumptions
- Factoring in the different perspectives of different stakeholder groups, e.g., sub-cultures, who will hold differing patterns of beliefs, behaviours, power and influence
- Developing cultural awareness for improvement both with staff and the middle- and top-management echelons
- Recognising that a positive, affirming culture is associated with positive clinical and organisational outcomes.

**Context** $\mathrm{OI}\;=\;\mathrm{ME}\;\cdot \;(w_{1}\mathrm{CL}\;+\;w_{2}\mathrm{CU}\;+\;w_{3}CX\;+\;w_{4}\mathrm{CN})$

We have shown that contexts differ. Local context must be taken into account in improvement activities. Strategies and tactics include:

- Acknowledging that context matters, is determinant of many workplace features, and operates at differing levels; from the core of care, through micro, meso and macro levels
- Appreciating that it is strategically necessary to operate at different context levels
- Working on understanding the environmental factors which shape the properties of a particular setting
- Apprehending that what works in one setting will not necessarily work, or will work differently, in another; change and improvement experiences need to be tailored to local circumstances.

**Connections** $\mathrm{OI}\;=\;\mathrm{ME}\;\cdot \;(w_{1}\mathrm{CL}\;+\;w_{2}\mathrm{CU}\;+\;w_{3}\mathrm{CX}\;+\;w_{4}CN)$

Connections, inter-dependencies and inter-relationships matter and must be understood in any improvement program striving to strengthen care. Strategies and tactics include:

- Knowing that at bedrock, all improvement involves activating others to do things differently; focusing on connections between people and agents is essential
- Understanding that it is not useful to construe leadership as a set of mechanistic operations or technical skills but as a mobilisation of stakeholders and their relationships, including dealing with power dynamics, emotions, incentives and motivations
- Appreciating deeply that trite aphorisms such as “people are our greatest asset” are insufficient; valuing staff rather than simply advocating for it is critical
- Recognising that genuinely working on collaboration, networking and building relationships is likely to pay improvement dividends, often handsomely, and creates a store of social capital that can be spent later.

All-in-all, in bringing these four sets of activities together, focusing on the complexity, culture, context and connections, it may be possible to create a force multiplier effect. No-one said change was easy without major effort and a considered strategic approach. We believe the Four Cs model is fundamental to profound improvement, in the ever-challenging efforts required to strengthen health systems

## Conclusion

### Four essential preconditions for improvement

We have articulated in the previous section that the improvement task is a suite of high-value activities, expressed as strategies and tactics which are at the heart of what a healthcare system or organisation can become if the intention is to genuinely overhaul healthcare using our Four Cs constructs. This is immensely hard, but is likely to yield more far-reaching results than merely skating over the top of change.

Improvement of care, then, is a four-element enhancement task: improving the core of care and its context, taking into account cultural attributes and connections, while apprehending the complexity of the organisation and the task, and focused on connections.

**Figure 6:**
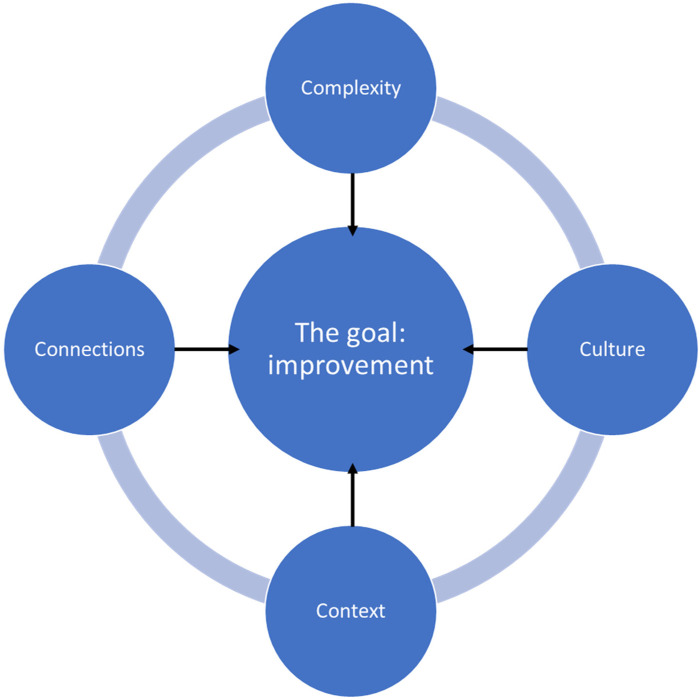
Synergies between complexity, culture, context and connections to improve care. Source: Authors’ conceptualisation.

Improvement requires collective efficacy, leadership and agency to move things forward. Thus, connected people integrated though productive cultures and positive environmental contexts can give direction to the social energy they have through a shared improvement goal, within the complex adaptive system of healthcare. At bottom, we are arguing for something extremely challenging but at a high level, is easy to diagram: Figure 6.

### How the Four Cs model relates to established frameworks

Several established implementation and improvement frameworks address elements of the Four Cs. Our intent is not to replace these approaches, but to offer an integrative ‘architect's view’ that helps teams coordinate them in practice. For example, the Four Cs maps to the Consolidated Framework for Implementation Research domains (70) (e.g., intervention complexity; inner setting culture; outer setting context; networks and communications) while making the need for coordinated design across the domains explicit It aligns with i-PARIHS through its emphasis on context and facilitation, while foregrounding relational ‘connections’ and coordination as a distinct design task (71, 72). It complements Normalization Process Theory by emphasising the work of embedding practices (coherence, cognitive participation, collective action and reflexive monitoring) and by suggesting where cultural and contextual constraints can impede that work (73). Finally, behaviour change approaches (COM-B, Behaviour Change Wheel and BCT taxonomy) can be used by ‘improvement architects’ to select and tailor techniques, mindful that capability, opportunity and motivation are shaped by culture, context and connections in complex systems (74, 75).

### Recommendations

We have presented the Four Cs model and argued that it supports a conceptual shift from ‘change agents’ to ‘improvement architects’—people who intentionally design, coordinate and adapt improvement efforts with Complexity, Cultures, Contexts and Connections in view. If the goal is deep, sustained improvement rather than more structural or cosmetic change, then we recommend leaders, managers, clinicians and policymakers should diagnose each C explicitly, align actions across levels, and invest in relational coordination and co-design so that improvement work fits the system's dynamics and lived realities. The model is offered as a practical heuristic rather than a measurable equation; its value lies in sharpening sensemaking and guiding design choices. Next steps include empirical evaluation through comparative case studies, realist-informed analyses and prospective testing of facilitation and coordination strategies that operationalise the Four Cs, alongside development of usable tools (e.g., diagnostic prompts and planning templates). We acknowledge that this paper is a narrative, conceptually synthesised perspective and the Four Cs will benefit from further refinement and validation in diverse settings.

## Authors’ information

JB has been a health systems researcher for over 30 years with specific interests in patient safety, complexity science and implementation science. RM has had a similarly extensively background to JB, and so has HP. RM's work ranges across health economics, culture and systems improvement. HP is a medical sociologist of many years standing. SW is a safety science expert with strong interest in resilience, patient safety and quality care.
